# Protocol optimization for callus induction and shoot regeneration of Ethiopian rice varieties (*Oryza sativa* L.)

**DOI:** 10.1186/s12896-025-00981-7

**Published:** 2025-05-24

**Authors:** Teslim Yimam, Mereme Abide, Solomon Benor, Demisachew Guadie

**Affiliations:** 1 Department of Biotechnology, College of Natural and Applied Science, Addis Ababa Science and Technology University, Addis Ababa, Ethiopia; 2 Department of Plant Biology and Biodiversity Management, College of Natural and Computational Sciences, Addis Ababa University, Addis Ababa, Ethiopia; 3 Department of Industrial Engineering, Faculty of Engineering and the Built Environment, Tshwane University of Technology, Pretoria, South Africa; 4 Institute of Biotechnology, Addis Ababa University, Addis Ababa, Ethiopia

**Keywords:** -2, 4-D, Callus, Genome editing, Kinetin, Shaga, Transformation, X-Jigna

## Abstract

Rice is the primary food source for the majority of the global population. In African countries, rice is considered a critical cereal crop essential for food security. Rice consumption in Ethiopia has been increasing from year to year, and the demand for rice food is not fulfilled by domestic production, the Ethiopian government imports rice products from Asia. Therefore increasing the production of rice in terms of quantity and quality through modern technology is a recent priority and strategy in the country. This study was, therefore, designed to optimize the protocol for callus induction and shoot regeneration of two Ethiopian rice (X-Jigna and Shaga) varieties. Three media types (LS, N6, and MS) supplemented with different concentrations of 2, 4-D were used for callus induction. The maximum callus induction was observed on MS-media supplemented with 2.5 mg/L for X-Jigna and Shaga varieties, respectively. The lowest callus induction frequencies were recorded on N6-media supplemented with 1.5 mg/L 2, 4-D for both varieties. The highest shoot regeneration for X-Jigna (80.67%) and Shaga (72%) was recorded on MS-media containing a combination of kinetin (2 mg/L) and Naphtalin Acetic Acid (0.2 mg/L). The survival rate of acclimatized plantlets was 100% and 66.6% for X-Jigna and Shaga, respectively. In conclusion, this study is distinctive as, to our knowledge, no other research has addressed the optimization of protocols for the Ethiopian rice varieties, serving as a foundational element for future investigations into rice transformation and breeding in Ethiopia.

**Supplementary Information:**

The online version contains supplementary material available at 10.1186/s12896-025-00981-7.

## Introduction

Rice is the world’s primary food crop, serving as a vital energy source for half of the global population, particularly in Asia, where it provides 50–80% of the daily calorie intake for over three billion people [19]. In Africa, rice is a strategic cereal crop for food security, with its consumption growing faster than that of other crops [13]. However, the demand for rice in Africa is outpacing supply, leading to increased imports each year to meet the continent’s rapidly increasing consumption needs [14].

The introduction and cultivation of rice is a relatively recent development in Ethiopia. According to various reports, rice was introduced in the 1970s and initially cultivated on a small scale [6, 18]. Nevertheless, the area dedicated to rice cultivation and its overall production has been steadily increasing [18]. A report by the Ministry of Agriculture and Rural Development [9] over 39 million hectares are suitable for rice production. Today, rice has been included in the list of Ethiopia’s National Food Security Crops, alongside wheat, maize, and tef. It has become the second most productive cereal crop after maize, contributing significantly to food and nutritional security, socio-economic development, income generation, and poverty reduction in the country [8, 9].

After the declaration of the rice as “Crop of Millennium” in 2007 the total consumption has grown in Ethiopia which is in line with the trend of other African countries consumption [15]. However; the area covered by rice cultivation and the production potential is not matched and like the other African countries Ethiopia also imports rice. According to the report by Alemu and Thompson [2], in 2018, Ethiopia imported 300,000 tons of rice. Now the government of Ethiopia and the minister of Agriculture have a special emphasis on rice R & D; because of the high need for rice for different purposes like preparing injera; bread and traditional alcoholic drinks. Different research centers introduce and release new rice varieties to the farmers that are biotic and abiotic resistant like X-Jigna and Shaga. X-Jigna is the most popular rice variety among farmers in Ethiopia, known for its broad adaptability to diverse rice-growing conditions. It was first introduced to the country in the 1970s with technical support from North Korean experts [18]. In contrast, the Shaga variety was introduced to Ethiopia in 2017 from the Africa Rice Center due to its exceptional cold tolerance and superior yield compared to the best-performing varieties, including X-Jigna, WAB 56–104, and Ediget [1]. However, these two varieties fail to fully meet farmers’ needs and demands. To address this gap, it is essential to utilize modern biotechnological tools such as plant tissue culture, genetic engineering, molecular diversity analysis, and genome editing. Therefore, the main objective of this study is protocol optimization for the two Ethiopian rice varieties X-Jigna and Shaga for callus and shoot regeneration response. It is a prerequisite for downstream biotechnological application tools, such as genetic transformation and genome editing.

## Materials and methods

The study was conducted in the Plant Tissue Culture Laboratory, Department of Biotechnology, Addis Ababa Science and Technology University, Ethiopia. The laboratory study was conducted from September 2022 to June 2023.

### Plant materials and sterilization

Two Ethiopian lowland rainfed rice (*Oryza sativa* L.) varieties X-Jigna and Shaga were used for the study. Seeds of the two varieties were obtained from Fogera National Rice Research and Training Center, Ethiopia. The matured seeds were de-husked manually using mortar and pestle, and surface sterilized with 75% alcohol (v/v) under a laminar airflow cabinet for two minutes. After discarding the alcohol, the seeds were further treated with 1% sodium hypochlorite having 1–2 drops of tween 20 for 15 min with shacking then repeat treating the seeds with 1% sodium hypochlorite for 15 min by shacking. Finally, properly sterilized seeds were rinsed 3–4 times using sterile distilled water and dried on sterilized filter paper before inoculation.

### Callus induction

The sterilized and dried seeds of X-Jigna and Shaga were inoculated into three media types (MS (Murashige and Skoog), N6 (Chu N6 media), and LS (Linsmaier and Skoog) basal media supplemented with 2, 4-D (1.5, 2.0, 2.5 and 3.0 mg/L) concentrations for induction of embryogenic calli. 3% sucrose was added to the media as a carbon source and 0.8% agar was used as a solidifying agent. The pH of each medium was adjusted to 5.8-6.0 before adding the solidifying agent using 1 N HCl and 1 N NaOH. The culture medium was sterilized using an autoclave at 121^o^C and 15 psi (105 kPa) for 15 min. After autoclaving 20–25 ml of the media was poured into Petri plates in the laminar air flow cabinet to maintain sterility of the culture media. The sterilized seeds were inoculated in the solidified media horizontally and sealed using parafilm. The cultured petri plates were placed and incubated in a growth chamber under 25 ± 2 °C temperature and a cycle of 16 h of light and 8 h of dark for 10–15 days. The experiment was replicated three times and about 150 seeds were inoculated for each media type and variety. The frequency of callus induction for each variety was recorded and the percentage of callus induction frequency was calculated as:

1 $$\begin{aligned}&Calli\:inductioin\:frequency\:\left(\%\right)\\ &\quad= \frac{Number\:of\:seeds\:produced\:calli\:}{Total\:no\:of\:seeds\:inoculated\:}\\&\quad*100\end{aligned}$$

### Shoot regeneration and rooting

Shoot regeneration was carried out by cutting 20–30 days old embryogenic calli. The calli were inoculated on MS basal media supplemented with 3% sucrose and different combinations and concentrations of plant growth hormone; i.e. Kinetin (1 mg/ L and 2 mg/ L) with NAA (0 mg/ L and 0.2 mg/ L) and BAP (1 mg/ L and 2 mg/ L) with NAA (0 mg/ L and 0.2 mg/ L). The pH of the media was adjusted to 5.8-6.0 before adding agar using 1 N HCl and 1 N NaOH. The media was melted after adding agar and poured into culture vessels, and sterilized at 121 ^o^C and 15 psi (105 kPa) for 15 min. In each experiment, 7 to 10 calli were inoculated to the media, and the vessels were sealed by parafilm. The culture vessels were maintained in a growth chamber at a temperature of 25 ± 2 ^°^C under a cycle of 16 h of light and 8 h of dark. The regenerated shoots were transferred to the same MS media composition for shoot elongation and were sub-cultured every two weeks. The number of regenerated shoots was recorded and calculated using Eq. 2 below

2 $$\begin{aligned}&Callus\:regeneration\:frequency\:\left(\%\right)\\&\quad=\frac{Number\:of\:calli\:produced\:shoots\:}{Total\:no\:of\:calli\:inoculated\:}\\&\quad*100\end{aligned}$$

Regenerated shoots were subjected to rooting by transferring into MS basal media without any plant growth regulators for one week.

### Acclimatization

In vitro regenerated plantlets with well-developed and healthy root systems were gently removed from the culture vessel and their roots were washed under running tap water to remove the agar. The plantlets were planted into pots containing a mixture of red soil, sand, and vermiculite (1:1:1) ratio. The pots were covered with polythene bags with semi-aeration to prevent excessive evapotranspiration and slowly adapt the plantlets to the external environment. The bags were removed after a week. The pots were watered every day and the survival rate of the acclimatized plantlets was recorded and the percentage was calculated as follows:

3 $$\begin{aligned}& Survived\:plantlets\:\left(\%\right)\\&\quad =\frac{Number\:of\:survived\:shoot}{Total\:no\:of\:acclimatized\:rooted\:shoots\:}\\&\quad*100\:\end{aligned}$$

### Statistical analysis

Callus induction and plantlet regeneration frequencies were calculated in percentage. The data was subjected to analysis of variance (ANOVA) using SPSS (version 30.0.0.0 (172)). The significant difference between means of different treatment concentrations and combinations was checked by using Fisher’s Least Significant Difference (LSD) test at p<0.05.

## Results

The effect of different types of basal media (MS, LS, and N6) and 2, 4-D concentration were tested for callus induction response. Shoot regeneration, multiplication, and root formation were also tested on MS-basal media supplemented with different concentrations and combinations of BAP, Kinetin, and NAA for both X-Jigna and Shaga Rice varieties.

### Effect of basal media on callus induction

The effect of MS, LS, and N6 basal media supplemented with different concentrations of 2, 4-D for callus induction frequency (CIF) response were tested and the result was calculated in percentage using Eq. (1) and significance was tested by ANOVA using SPSS (*p* < 0.05).

**Fig. 1:**
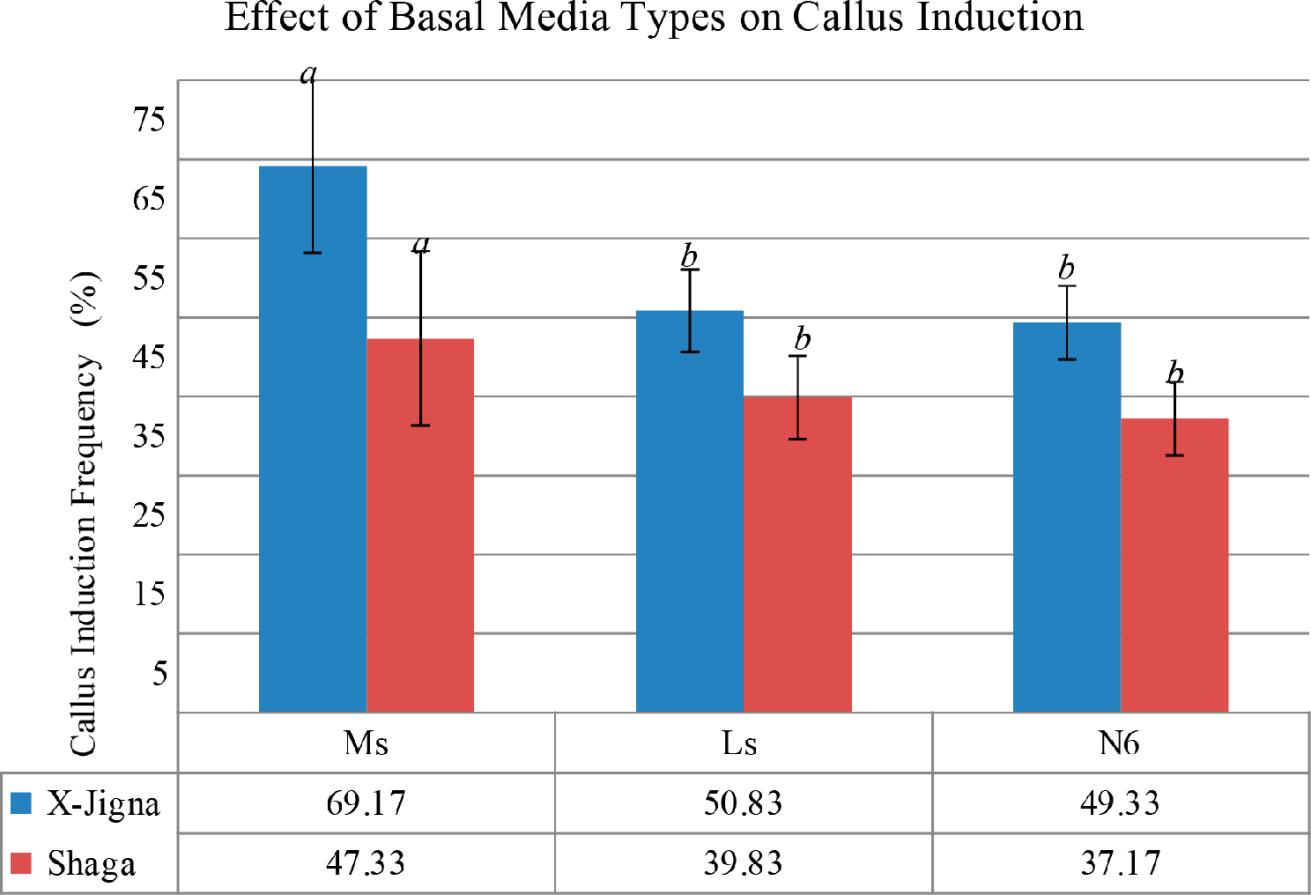
Callus induction frequency for two Ethiopian rice varieties (X-Jigna and Shaga) cultured on different basal media MS, N6, and LS media, least significant Difference test *p*<0.05

The seeds inoculated on MS media with different concentrations of 2, 4-D (1.5 mg/L, 2.0 mg/L, 2.5 mg/L, and 3.0 mg/L) showed better response for both X-Jigna and Shaga i.e. 69.17% and 47.33% CIF respectively. Whereas, seeds cultured on N6 media showed the lowest percentage of CIF 49.33% and 37.17% for X-Jigna and Shaga rice varieties respectively (see Fig. 1). However, there is no significant difference between the three types of basal media.

### Effect of 2, 4-D concentration on callus induction

**Fig. 2:**
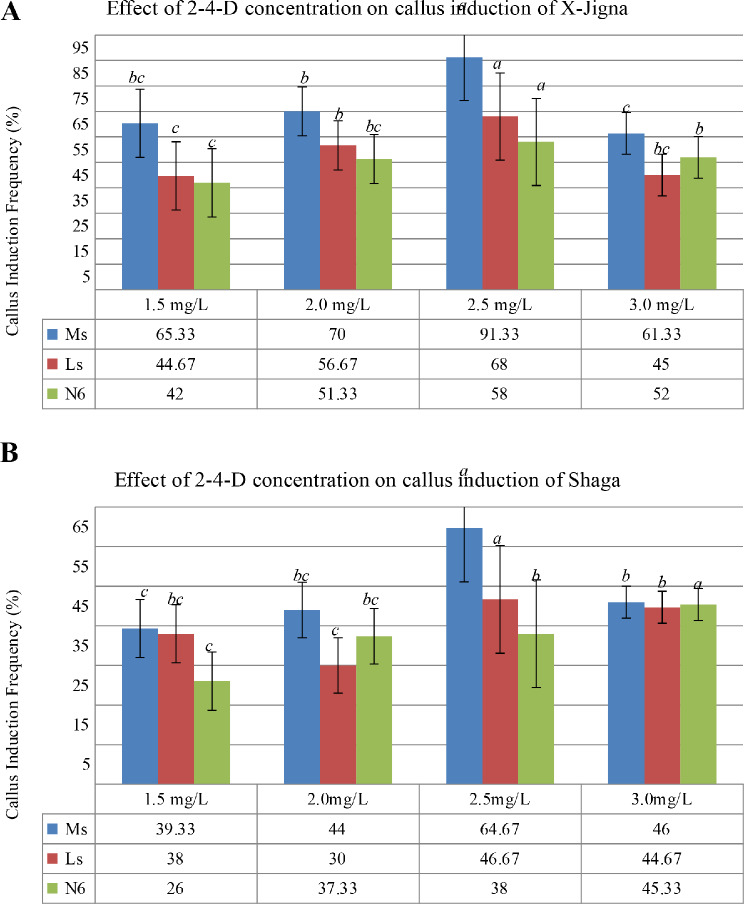
Callus induction frequency (%) for the two Ethiopian rice varieties (**A**) X-Jigna and (**B**) Shaga supplemented with different concentrations of 2, 4-D. Different letters denote significant differences at *p*<0.05

The frequency of callus induction on the three types of basal media supplemented with different concentrations of 2, 4-D (1.5 mg/L, 2.0 mg/L, 2.5 mg/L, and 3.0 mg/L) was varied and significant at *p* < 0.05 for both X-Jigna and Shaga (supplementary 1). The highest callus induction frequency for X-Jigna (91.33%) and Shaga (64.67%) were observed on MS medium supplemented with 2.5.mg/L 2, 4-D concentration respectively. Minimum CIF 42% and 26% were recorded on N6 media containing 1.5 mg/L 2, 4-D for X-Jigna and Shaga respectively (see Fig. 2A and B).

### Shoot proliferation from calli

Calli with better quality from each basal media were used for shoot proliferation frequency optimization. The result of this study showed that the percentage of regenerated shoots for both X-Jigna and Shaga rice varieties did not vary significantly (Table 1). Therefore, shoot regeneration of X-Jigna and Shaga rice varieties did not respond differently.

**Table 1:** Analysis of variance (ANOVA) results for shoot regeneration of X-Jigna and Shaga (*p* < 0.05)

Mean regeneration	df	Sum of squares	Mean square	F	Sig.
Between Groups	1	36.750	36.750	0.686	0.427
Within Groups	10	535.352	53.535		
Total	11	572.102			

**Fig. 3:**
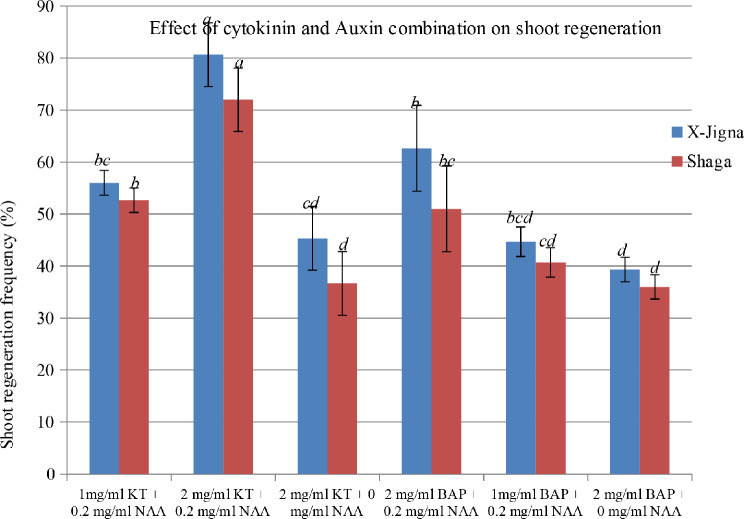
Shoot regeneration frequency from callus in response to various concentrations and combinations of KT, BAP, and NAA on MS media for both X-Jigna and Shaga Different letters denote significant differences at *p*<0.05

Among the various combinations and concentrations of cytokinin and auxin the maximum shoot regeneration for X-Jigna (80.67%) and Shaga (72%) were recorded on MS media supplemented with a combination of 2 mg/L Kinetin (KT) and 0.2 mg/L Naphtalin Acetic Acid (NAA) (see Fig. 3). Also MS media containing a combination of BAP (2 mg/L) and NAA (0.2 mg/L) showed relatively good regeneration frequency as compared with the other treatments. The response for shoot regeneration on MS media which was supplemented KT or BAP alone showed minimum shoot regeneration frequency for both X-Jigna and Shaga rice varieties (Fig. 3).

**Fig. 4:**
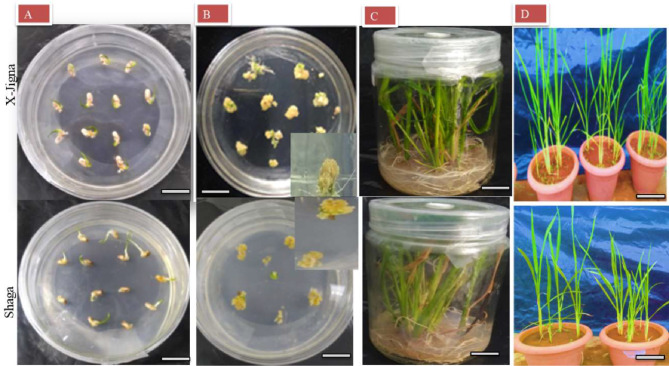
Establishment of tissue culture protocol for the two Ethiopian rice varieties X-Jigna and shaga respectively (**A**) embryogenic calli induction from seed explant on MS media supplemented with 2.5 mg/L 2, 4-D after five days of culture (**B**) matured and enlarged callus on MS media supplemented with 2.5 mg/L 2, 4-D after three weeks (**C**) regenerated plantlets from the induced calli on MS supplemented with 2 mg/L KT and 0.2 mg/L NAA (**D**) hardened plantlets of in vitro regenerated and rooted plantlets after one month. Bars = 3 cm

Overall MS media showed better response for both callus induction and also shoot regeneration of X-Jigna and Shaga (see Fig. 4).

### Rooting and acclimatization of rooted shoots

Root were formed in all regeneration MS media supplemented with a combination of cytokinin and auxin (1 mg/L KT + 0.2 mg/L NAA, 2 mg/L KT + 0.2 mg/L NAA, 1 mg/L BAP + 0.2 mg/L NAA and 2 mg/L BAP + 0.2 mg/L NAA) for both X-Jigna and Shaga rice varieties. Afterward, all rooted shoots were cultured in MS media without growth hormones for one week. The well-rooted shoots were then transferred to soil for acclimatization.

The plantlets with healthy and well-developed root systems were transferred to a mixture of soil and placed in the greenhouse. The maximum survival rates of the two rice varieties were 100% and 66.67% for X-Jigna and Shaga, respectively (Fig. 4d).

## Discussion

Crop improvement using biotechnological tools such as plant transformation genome editing and others mostly requires an efficient and effective protocol for in vitro callus induction and plantlet regeneration. The present study focused on protocol optimization for callus induction and plantlet regeneration of two Ethiopian rice varieties X-Jigna and Shaga. In this study, the frequency of callus induction and plantlet regeneration depends on the genotype of the rice variety, basal media types, and concentration of 2, 4-D, and other cytokinin and auxin plant growth hormones which is similar to the other studies reported on rice and also wheat [3, 7, 11].

The effect of 2, 4-D concentration in a callus induction media for different rice varieties was reported and agreed with the current study for X-Jigna and Shaga. In this finding at 2 mg/L and 2.5 mg/L 2, 4-D concentration the CIF for Shaga and X-Jigna were maximum that is consistence with previous reports [3, 4, 16]. According to Gao et al., [5] callus generation was significantly higher without the combination of any cytokinin. Conversely, the impact of 2,4-D concentration on various basal media types (MS, N6, and LS) tested in this experiment revealed differences in callus induction frequency for both rice varieties, which align with other studies on rice [7, 11, 17]. Maximum CIF was obtained on MS basal media. However, both X-Jigna and Shaga showed minimum CIF on LS basal media, which is similar to the previous report by [3].

According to Lee et al. (2002), the effect of cytokinin and auxin on promoting shoot regeneration is due to interaction mechanisms like synergetic, antagonistic, and additive effects. Meanwhile, in the present study, the combination of KT and BAP with NAA resulted in the proliferation of maximum shoot for the two Ethiopian rice varieties X-Jigna and Shaga, consistent with other previous reports by [3, 10]. Noor et al., [12] also suggested that increasing the concentration of either KT or BAP increased regeneration frequency.

## Conclusion

The study concluded that from the different types of basal media, the seed cultured in MS-media showed higher callus induction frequency for X-Jigna and Shaga varieties. On the other hand, the concentration of growth regulator also affected the callus induction frequency in all types of basal media. The other observation from this study was that the combination of cytokinin (high concentration) and Auxin (low concentration) showed higher shoot regeneration from the callus for the two varieties. Rice variety X-Jigna performed better than Shaga in this protocol optimization study, such as in callus induction, shoot regeneration, and also in hardening.

## Electronic supplementary material

Below is the link to the electronic supplementary material.

Supplementary Material 1

## Data Availability

The dataset generated and/or analyzed during the study will provide from the corresponding author upon the request.

## Declarations

I declare that this study is my original work and has not been submitted to any other institution anywhere for the award of any academic degree, diploma, or certificate. All sources of materials used for the study have been duly acknowledged.

## Abbreviations

BAP: 6-Benzylamino purine
CIF: Callus induction frequency, 2
4-D 2: 4-Dichlorophenoxyacetic acid
KT: Kinetin
LS: Linsmaier and Skoog
MS: Murashige and Skoog
N6: Chu N6 media
NAA: Naphtalin Acetic Acid
