# Supplementary material for: Protocol optimization for callus induction and shoot regeneration of Ethiopian rice varieties (Oryza sativa L.)
Source: BMC Biotechnol. 2025 May 24;25:43. doi: 10.1186/s12896-025-00981-7 (PMC12103809; doi:10.1186/s12896-025-00981-7)
Supplement: Supplementary file 1 — Supplementary Material 1 [file 12896_2025_981_MOESM1_ESM.pdf]

Oneway

Notes

Output Created		01-JAN-2025 12:12:53
Comments		
Input	Active Dataset	DataSet0
	Filter	<none>
	Weight	<none>
	Split File	<none>
	N of Rows in Working Data File	24
Missing Value Handling	Definition of Missing	User-defined missing values are treated as missing.
	Cases Used	Statistics for each analysis are based on cases with no missing data for any variable in the analysis.
Syntax		ONEWAY mean_callus_Indu BY Genotype /ES=OVERALL /STATISTICS DESCRIPTIVES /MISSING ANALYSIS /CRITERIA=CILEVEL (0.95) /POSTHOC=LSD ALPHA (0.05).
Resources	Processor Time	00:00:00.03
	Elapsed Time	00:00:00.04

[DataSet0]

Warnings

Post hoc tests are not performed for mean_callus_Indu because there are fewer than three groups.

Descriptives

mean_callus_Indu

	N	Mean	Std. Deviation	Std. Error	95% Confidence Interval for Mean		Minimum	Maximum
					Lower Bound	Upper Bound		
X_Jigna	12	28.3889	6.71172	1.93751	24.1245	32.6533	21.00	45.67
Shaga	12	20.3611	5.06415	1.46189	17.1435	23.5787	13.00	32.33
Total	24	24.3750	7.11487	1.45232	21.3707	27.3793	13.00	45.67

ANOVA

mean_callus_Indu

	Sum of Squares	df	Mean Square	F	Sig.
Between Groups	386.671	1	386.671	10.939	.003
Within Groups	777.620	22	35.346		
Total	1164.292	23			

ANOVA Effect Sizes[a]

		Point Estimate	95% Confidence Interval	
			Lower	Upper
mean_callus_Indu	Eta-squared	.332	.047	.554
	Epsilon-squared	.302	.004	.534
	Omega-squared Fixed-effect	.293	.004	.524
	Omega-squared Random-effect	.293	.004	.524

a. Eta-squared and Epsilon-squared are estimated based on the fixed-effect model.

Oneway

Notes

Output Created		01-JAN-2025 12:13:37
Comments		
Input	Active Dataset	DataSet0
	Filter	<none>
	Weight	<none>
	Split File	<none>
	N of Rows in Working Data File	24
Missing Value Handling	Definition of Missing	User-defined missing values are treated as missing.
	Cases Used	Statistics for each analysis are based on cases with no missing data for any variable in the analysis.
Syntax		ONEWAY mean_callus_Indu BY Basal_media /ES=OVERALL /STATISTICS DESCRIPTIVES /MISSING ANALYSIS /CRITERIA=CILEVEL (0.95) /POSTHOC=LSD ALPHA (0.05).
Resources	Processor Time	00:00:00.02
	Elapsed Time	00:00:00.02

Descriptives

mean_callus_Indu

	N	Mean	Std. Deviation	Std. Error	95% Confidence Interval for Mean		Minimum
					Lower Bound	Upper Bound	
MS	8	29.1250	8.52808	3.01513	21.9953	36.2547	19.67
N6	8	21.7500	4.67940	1.65442	17.8379	25.6621	13.00
LS	8	22.2500	5.73419	2.02734	17.4561	27.0439	14.67
Total	24	24.3750	7.11487	1.45232	21.3707	27.3793	13.00

Descriptives

mean_callus_Indu

	Maximum
MS	45.67
N6	29.00
LS	32.67
Total	45.67

ANOVA

mean_callus_Indu

	Sum of Squares	df	Mean Square	F	Sig.
Between Groups	271.750	2	135.875	3.197	.061
Within Groups	892.542	21	42.502		
Total	1164.292	23			

ANOVA Effect Sizes[a,b]

		Point Estimate	95% Confidence Interval	
			Lower	Upper
mean_callus_Indu	Eta-squared	.233	.000	.453
	Epsilon-squared	.160	-.095	.401
	Omega-squared Fixed-effect	.155	-.091	.391
	Omega-squared Random-effect	.084	-.043	.243

a. Eta-squared and Epsilon-squared are estimated based on the fixed-effect model.

b. Negative but less biased estimates are retained, not rounded to zero.

Post Hoc Tests

Multiple Comparisons

Dependent Variable: mean_callus_Indu
LSD

(I) Basal_media	(J) Basal_media	Mean Difference (I-J)	Std. Error	Sig.	95% Confidence Interval Lower Bound	Upper Bound
MS	N6	7.37500*	3.25968	.034	.5961	14.1539
	LS	6.87500*	3.25968	.047	.0961	13.6539
N6	MS	-7.37500*	3.25968	.034	-14.1539	-.5961
	LS	-.50000	3.25968	.880	-7.2789	6.2789
LS	MS	-6.87500*	3.25968	.047	-13.6539	-.0961
	N6	.50000	3.25968	.880	-6.2789	7.2789

*. The mean difference is significant at the 0.05 level.

Oneway

Notes

Output Created		01-JAN-2025 12:14:56
Comments		
Input	Active Dataset	DataSet0
	Filter	<none>
	Weight	<none>
	Split File	<none>
	N of Rows in Working Data File	24
Missing Value Handling	Definition of Missing	User-defined missing values are treated as missing.
	Cases Used	Statistics for each analysis are based on cases with no missing data for any variable in the analysis.
Syntax		ONEWAY mean_callus_Indu BY Horm_con<br>/ES=OVERALL<br>/STATISTICS DESCRIPTIVES<br>/MISSING ANALYSIS<br>/CRITERIA=CILEVEL (0.95)<br>/POSTHOC=LSD ALPHA (0.05).
Resources	Processor Time	00:00:00.03
	Elapsed Time	00:00:00.03

Descriptives

mean_callus_Indu

	N	Mean	Std. Deviation	Std. Error	95% Confidence Interval for Mean		Minimum
					Lower Bound	Upper Bound	
1.50	6	20.1111	6.06508	2.47606	13.7462	26.4760	13.00
2.00	6	22.8333	5.70672	2.32976	16.8445	28.8222	15.00
2.50	6	30.8889	8.45555	3.45196	22.0153	39.7624	22.33
3.00	6	23.6667	3.97772	1.62390	19.4923	27.8410	20.00
Total	24	24.3750	7.11487	1.45232	21.3707	27.3793	13.00

Descriptives

mean_callus_Indu

	Maximum
1.50	30.00
2.00	31.67
2.50	45.67
3.00	31.00
Total	45.67

ANOVA

mean_callus_Indu

	Sum of Squares	df	Mean Square	F	Sig.
Between Groups	380.940	3	126.980	3.242	.044
Within Groups	783.352	20	39.168		
Total	1164.292	23			

ANOVA Effect Sizes[a,b]

		Point Estimate	95% Confidence Interval	
			Lower	Upper
mean_callus_Indu	Eta-squared	.327	.000	.513
	Epsilon-squared	.226	-.150	.440
	Omega-squared Fixed-effect	.219	-.143	.430
	Omega-squared Random-effect	.085	-.043	.201

a. Eta-squared and Epsilon-squared are estimated based on the fixed-effect model.

b. Negative but less biased estimates are retained, not rounded to zero.

Post Hoc Tests

Multiple Comparisons

Dependent Variable: mean_callus_Indu

LSD

(I) Horm_con	(J) Horm_con	Mean Difference (I-J)	Std. Error	Sig.	95% Confidence Interval	
					Lower Bound	Upper Bound
1.50	2.00	-2.72222	3.61329	.460	-10.2594	4.8150
	2.50	-10.77778*	3.61329	.007	-18.3150	-3.2406
	3.00	-3.55556	3.61329	.337	-11.0927	3.9816
2.00	1.50	2.72222	3.61329	.460	-4.8150	10.2594
	2.50	-8.05556*	3.61329	.037	-15.5927	-.5184
	3.00	-.83333	3.61329	.820	-8.3705	6.7039
2.50	1.50	10.77778*	3.61329	.007	3.2406	18.3150
	2.00	8.05556*	3.61329	.037	.5184	15.5927
	3.00	7.22222	3.61329	.059	-.3150	14.7594
3.00	1.50	3.55556	3.61329	.337	-3.9816	11.0927
	2.00	.83333	3.61329	.820	-6.7039	8.3705
	2.50	-7.22222	3.61329	.059	-14.7594	.3150

*. The mean difference is significant at the 0.05 level.
